# Combined Effects of Environmental Metals and Physiological Stress on Lipid Dysregulation

**DOI:** 10.3390/medsci12040051

**Published:** 2024-10-02

**Authors:** Emmanuel Obeng-Gyasi, Yvonne R. Ford

**Affiliations:** 1 Department of Built Environment, North Carolina A&T State University, Greensboro, NC 27411, USA; 2 Environmental Health and Disease Laboratory, North Carolina A&T State University, Greensboro, NC 27411, USA; 3 School of Nursing, North Carolina A&T State University, Greensboro, NC 27411, USA

**Keywords:** metals, stress, cardiovascular, mixtures, exposome

## Abstract

Background: Cardiovascular diseases (CVD) are a leading cause of mortality worldwide, influenced by genetic, environmental, and behavioral factors. This study examines the relationship between heavy metal exposure, chronic physiological stress (allostatic load), and lipid profiles, which are markers of CVD risk, using data from the National Health and Nutrition Examination Survey (NHANES) 2017–2018. Methods: We utilized structural equation modeling (SEM) to explore the associations between blood levels of lead, cadmium, allostatic load (AL), and lipid measures (low-density lipoprotein (LDL), high-density lipoprotein (HDL), and triglycerides). The AL index was derived from cardiovascular, inflammatory, and metabolic biomarkers and categorized into quartiles to identify high-risk individuals, with an index out of 10 subsequently developed. Results: The SEM analysis revealed that both heavy metal exposure and allostatic load are significantly associated with lipid profiles. Higher levels of lead and cadmium were associated with increased LDL and triglycerides, while higher AL scores were linked to increased LDL and triglycerides and decreased HDL levels. Age was also a significant factor, showing positive correlations with LDL and triglycerides, and a negative correlation with HDL. Conclusions: This study underscores the multifactorial nature of CVD, highlighting the combined impact of environmental pollutants and physiological stress on lipid dysregulation. These findings suggest the need for integrated public health strategies that address both environmental exposures and chronic stress to mitigate cardiovascular risk. Further research is warranted to explore the underlying mechanisms and develop targeted interventions.

## 1. Introduction

Cardiovascular diseases (CVD) remain a leading cause of mortality worldwide, driven by a complex interplay of genetic, environmental, and behavioral factors [1]. Lipid markers, such as LDL, triglycerides, and HDL, are key indicators of CVD risk, as they reflect the levels of fats in the blood that can contribute to the development of atherosclerosis and other heart-related conditions. The role of environmental pollutants, such as heavy metals, in exacerbating cardiovascular risk has garnered increasing attention [2,3]. Lead and cadmium are common environmental contaminants with overlapping exposure sources. Lead is often found in lead-based paints, contaminated soil, water from lead pipes, and industrial emissions, while cadmium exposure commonly occurs through tobacco smoke and food grown in contaminated soil [4,5]. Both metals co-occur near industrial sites, smelting facilities, and mining operations, contaminating soil, water, and air. Airborne particulates from industrial processes and waste incineration, as well as household dust in older buildings, further contribute to co-exposure. These shared pathways underscore the importance of studying their combined health effects [6,7].

Lead in blood typically has a half-life of about 28 days, after which it is redistributed to bones, where it can be stored for decades and released back into the bloodstream during stress, illness, or bone remodeling [8]. Cadmium, on the other hand, has a much longer half-life in blood, approximately three to four months, due to its accumulation in organs such as the kidneys and liver, from where it is slowly released. These half-lives indicate that blood levels can provide insight into both recent and cumulative exposure [9].

Exposure to both metals primarily occurs via inhalation and ingestion, with inhalation being more common in occupational settings, such as smelting and manufacturing, and ingestion being more common from contaminated food, water, and soil. Dermal absorption is generally less significant for both metals, though it can contribute in specific cases, such as handling contaminated materials without protective equipment. Ingestion and inhalation are the most common exposure routes for the general population, while occupational exposure typically involves inhalation [9].

Lead and cadmium, pervasive environmental contaminants, have been implicated in a range of adverse health outcomes, including hypertension, atherosclerosis, and dyslipidemia [10,11,12]. As of 28 October 2021, the CDC set the blood lead reference value (BLRV) at 3.5 µg/dL to identify children with elevated levels. Although no safe threshold exists, blood lead levels above 5 µg/dL in adults are generally considered toxic and are linked to adverse health effects such as hypertension and cognitive decline [13,14].

For cadmium, normal blood concentrations are less than 5.0 ppb, with typical levels ranging from 0.5 to 2.0 ppb [15]. Acute toxicity is observed when blood cadmium exceeds 50 ppb. Smokers generally have blood cadmium levels of 1–4 ppb, significantly higher than non-smokers, whose levels are usually below 0.5 ppb [16]. Despite extensive research, the precise mechanisms through which these metals influence cardiovascular health are not fully understood [17].

In parallel, the concept of allostatic load (AL)—the cumulative physiological burden exacted on the body by chronic stress—has emerged as a critical factor in understanding the etiology of CVD [18,19]. AL encompasses dysfunction across multiple biological systems, including cardiovascular, inflammatory, and metabolic pathways [20]. Elevated AL scores, reflecting heightened physiological stress, are associated with increased risk for a range of chronic diseases, including CVD.

The integration of environmental and physiological stressors provides a more comprehensive understanding of CVD risk [21]. This study examines the role of lead and cadmium levels, as well as AL, with key lipid measures: low-density lipoprotein (LDL), high-density lipoprotein (HDL), and triglycerides.

Our study leverages structural equation modeling, a comprehensive statistical technique that allows for the examination of complex relationships among observed and latent variables, enabling the testing of theoretical models that specify how variables interact with each other [22]. By employing structural equation modeling, we aim to elucidate the complex associations between these variables and identify potential statistical relationships through which environmental and physiological stressors contribute to lipid dysregulation and, ultimately, cardiovascular risk. Understanding these relationships is essential for developing targeted interventions to mitigate the impact of environmental pollutants and chronic stress on cardiovascular health. This study contributes to the growing body of literature on the multifactorial nature of CVD, highlighting the importance of addressing both environmental exposures and physiological stress in public health strategies.

## 2. Materials and Methods

### 2.1. Data Source

The data for this study were obtained from the National Health and Nutrition Examination Survey (NHANES) 2017–2018. NHANES is a research initiative aimed at evaluating the health and nutritional conditions of both adults and children across the United States. This study incorporates a combination of interviews and physical examinations. For the present analysis, we utilized data on serum lipid levels, including LDL, HDL, and triglycerides, along with relevant covariates.

### 2.2. Study Population

The analysis utilized participants from the NHANES 2017–2018 dataset who had complete data on serum LDL, HDL, triglycerides, lead, cadmium, allostatic load (AL), age, and other relevant covariates. The total study sample consisted of 9254 participants. Among these participants, HDL cholesterol measurements were available for 6738 people, while LDL cholesterol data was available for 2808 participants, and triglyceride measurements were available for 2834 participants. Lead exposure was assessed in 6884 participants, and cadmium exposure was measured in 7513 participants, reflecting varying levels of data availability across key variables.

### 2.3. Study Variables

This study includes the following key variables. The outcome variables are LDL cholesterol levels, serum triglyceride levels, and HDL cholesterol levels. The predictor variables in the analysis include blood lead levels, blood cadmium levels, and allostatic load (AL), which is a composite measure of physiological stress. Blood lead and cadmium levels were combined into a latent variable called “Metals”, representing the shared variance between these two environmental toxicants. This latent variable captures the common underlying exposure risk associated with both lead and cadmium, allowing for a more integrated assessment of their combined impact on health outcomes. Additionally, covariates include the participant’s age, which will be controlled for in the analyses to account for potential confounding effects. These variables are critical for understanding the relationship between the studied population’s environmental exposures, stress, and lipid profiles.

### 2.4. Operationalizing Allostatic Load

The allostatic load index was constructed as a cumulative measure of physiological dysfunction, based on previous research. It incorporates markers from the cardiovascular system (including SBP, DBP, triglycerides, HDL cholesterol, and total cholesterol), the inflammatory system (CRP), and the metabolic system (BMI, hemoglobin A1C, albumin, and creatinine clearance) [23,24]. The AL markers were categorized into quartiles according to their distribution in the dataset. For most markers, the top 25% was classified as high-risk. However, for albumin, creatinine clearance, and HDL cholesterol, the bottom 25% represented the high-risk group. Each participant scored 1 if they fell into the high-risk category for a given marker, or 0 if they were in the low-risk group. These scores were summed to create a total AL value out of 10. Details regarding the clinical and laboratory methods for marker collection and variable analysis have been outlined in previous studies.

### 2.5. Statistical Analysis

The statistical analysis was conducted using R version 4.2.3. Structural equation modeling (SEM) was employed to examine the relationships between heavy metal exposure (lead and cadmium), allostatic load, age, and lipid levels (LDL, triglycerides, and HDL). SEM allows for the estimation of complex relationships between observed and latent variables, accounting for measurement error and providing a comprehensive understanding of the direct and indirect effects [25,26].

### 2.6. Data Preparation

Continuous variables, including lead, cadmium, AL, age, and lipid measures, were standardized (z-scores) prior to analysis. This standardization facilitates the comparison of coefficients within the SEM framework.

#### 2.6.1. SEM Model Specification

Three separate structural equation models (SEM) were specified and estimated, each focusing on a different lipid measure—LDL, triglycerides, or HDL—as the outcome variable. In these models, the latent variable “Metals” was defined to capture the common variance between lead and cadmium levels. The measurement model for each SEM included this latent variable, which was measured by standardized levels of lead and cadmium. The structural model then involved regressing the respective outcome variable—either LDL, triglycerides, or HDL—on the latent variable metals, along with standardized AL and standardized age. This approach enabled a detailed examination of how environmental metal exposure, physiological stress, and age interact to influence lipid profiles.

#### 2.6.2. Model Estimation

The models were estimated using the lavaan package in R. Maximum Likelihood (ML) estimation was employed to fit the models. Model fit was assessed using the Chi-square test, Comparative Fit Index (CFI), Tucker–Lewis Index (TLI), Root Mean Square Error of Approximation (RMSEA), and Standardized Root Mean Square Residual (SRMR).

#### 2.6.3. Results Presentation

Parameter estimates, including path coefficients, standard errors, z-values, and *p*-values, were reported for each model. The significance of the relationships between the latent variables metals, AL, age, and lipid measures was interpreted based on these estimates.

All analyses were conducted in R version 4.2.3; a *p*-value < 0.05 was considered significant.

## 3. Results

Table 1 shows the survey-weighted mean levels and standard errors (SE) of key variables from the NHANES 2017–2018 dataset. The mean blood lead level was 1.07 µg/dL (SE = 0.03), and the mean blood cadmium level was 0.43 µg/L (SE = 0.02). The mean allostatic load (AL) score was 3.50 (SE = 0.06). For lipid measures, the mean LDL cholesterol level was 108.66 mg/dL (SE = 1.40), the mean triglyceride level was 102.37 mg/dL (SE = 1.94), and the mean HDL cholesterol level was 54.34 mg/dL (SE = 0.72). The mean age of the participants was 44.33 years (SE = 0.62).

**Figure 1:**
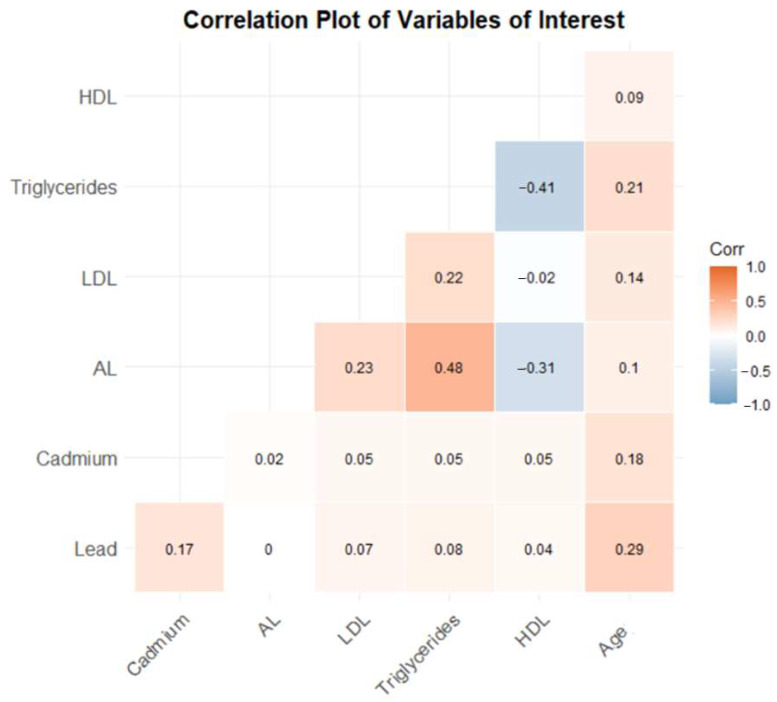
Spearman correlations among the lipid markers, allostatic load, and metals.

The correlation plot (Figure 1) illustrates the relationships between critical variables, including lead, cadmium, AL, LDL cholesterol, triglycerides, HDL cholesterol, and age, using NHANES 2017–2018 data. Lead and cadmium levels show a weak positive correlation (0.17). AL is moderately positively correlated with triglycerides (0.48) and weakly with LDL (0.23), but moderately negatively correlated with HDL (−0.31). LDL has weak positive correlations with AL (0.23) and triglycerides (0.22). Triglycerides and HDL are moderately negatively correlated (−0.41). Age exhibits weak positive correlations with LDL (0.14), triglycerides (0.10), and HDL (0.21). These findings underscore the interplay between environmental exposures, physiological stressors, and lipid levels.

The structural equation model examined the relationships between heavy metal exposure, physiological stress, age, and LDL cholesterol levels (Table 2), with adjustments made for age. The results are summarized below. The structural equation model analysis revealed that both heavy metal exposure (as captured by the latent variable metals) and allostatic load significantly contribute to variations in LDL cholesterol levels, even after adjusting for age. Specifically, higher levels of lead and cadmium were associated with increased LDL cholesterol. Similarly, greater physiological stress (allostatic load) and older age were linked to higher LDL cholesterol levels. These findings underscore the importance of considering environmental and physiological stressors in understanding cholesterol metabolism and cardiovascular risk.

The structural equation model examined the relationships between heavy metal exposure, physiological stress, age, and HDL cholesterol levels, with adjustments made for age. The results are summarized below.

The structural equation model analysis revealed that allostatic load and age significantly contribute to variations in HDL cholesterol levels (Table 3), even after adjusting for heavy metal exposure. Specifically, higher levels of allostatic load were associated with decreased HDL cholesterol, while older age was linked to increased HDL cholesterol levels. The direct effect of heavy metals (as captured by the latent variable metals) on HDL cholesterol was not statistically significant.

The structural equation model examined the relationships between heavy metal exposure, physiological stress, age, and triglyceride levels, with adjustments made for age. The results are summarized below.

The structural equation model analysis revealed that allostatic load and age significantly contribute to variations in triglyceride levels (Table 4), even after adjusting for heavy metal exposure. Specifically, higher levels of allostatic load and older age were associated with increased triglyceride levels. The direct effect of heavy metals (as captured by the latent variable metals) on triglyceride levels was not statistically significant.

## 4. Discussion

This study aimed to elucidate the complex interplay between environmental pollutants, physiological stress, and lipid profiles by analyzing data from the NHANES 2017–2018 cohort. The findings provide significant insights into how exposure to heavy metals, such as lead and cadmium, along with elevated AL, is associated with lipid dysregulation, which may influence CVD risk. We identified a clear association between metal exposure and lipid metabolism, consistent with findings reported in other studies [12,27]. Notably, both lead and cadmium have been implicated in adverse cardiovascular events such as myocardial infarction and ischemic stroke [11,18,19]. 

Our SEM results revealed that lead and cadmium are not the primary drivers of the latent “Metals” factor in triglyceride and HDL cholesterol models. 

Specifically, the SEM results revealed a significant impact of the latent “Metals” variable on LDL cholesterol but not on triglycerides or HDL cholesterol. This difference may be attributed to the distinct biological mechanisms regulating these lipids [17,28]. While all three are crucial components of lipid metabolism, they are influenced differently by environmental factors such as heavy metal exposure [17,29,30,31].

LDL cholesterol appears to be particularly sensitive to the effects of lead and cadmium, likely due to their roles in inducing oxidative stress [32]. This oxidative stress can disrupt the normal pathways of cholesterol synthesis and regulation, leading to elevated LDL levels. In contrast, triglycerides and HDL cholesterol may be regulated by pathways that are less directly affected by metal-induced oxidative stress, explaining the lack of a significant relationship in the SEM results. Additionally, the biological pathway linking metal exposure to LDL levels may be more specific and direct, whereas the pathways influencing triglycerides and HDL cholesterol are likely more complex and involve additional factors not captured by the metal variable [10,33]. 

Additionally, our results indicated that a higher AL, reflecting cumulative physiological stress, is significantly associated with adverse lipid profiles. Specifically, elevated AL scores were linked to increased LDL and triglycerides and reduced HDL. Similar findings from the Jackson Heart Study indicate that the burden of AL and its association with coronary heart disease is particularly pronounced in Black/African American populations [34,35,36]. This underscores the significant health disparities that exist, where undue stressors—often exacerbated by socio–economic and environmental factors—disproportionately affect these communities. Chronic stress, whether due to systemic inequities or other factors, can lead to dysregulation across multiple biological systems, contributing to lipid abnormalities.

The stress-induced hormonal changes, such as elevated cortisol levels, are known to adversely affect lipid metabolism, leading to higher LDL and triglyceride levels and lower HDL levels. These findings emphasize the importance of addressing racial and ethnic disparities in health, as the compounded effects of stress and structural inequities can lead to increased CVD risk in these populations.

Moreover, the impact of stress on lipid metabolism is compounded by other factors, such as aging, which was also found to be positively correlated with LDL and triglycerides and negatively correlated with HDL. As individuals age, various metabolic changes occur that can exacerbate lipid dysregulation and further elevate CVD risk [37,38]. Addressing these issues, particularly in vulnerable racial and ethnic groups, is crucial for reducing health disparities and improving cardiovascular outcomes.

Our findings highlight the critical role of environmental and physiological factors in the management and prevention of CVD. The exposome, which encompasses the totality of environmental exposures throughout a person’s life, is a significant contributor to the global burden of CVD. Munzel and colleagues further emphasize this by demonstrating how environmental exposures exacerbate key CVD risk factors, thereby amplifying the impact of environmental determinants of CVD on populations globally [21]. 

Public health strategies should focus on reducing environmental exposure to lead and cadmium through policies that enforce stricter regulations on industrial emissions and improve monitoring of environmental contaminants. It is also essential to refine and standardize health risk assessments for heavy metal contamination from various sources, including soil, water, and air. This comprehensive approach would better evaluate the bioavailability of these contaminants, ensuring that public health interventions effectively minimize exposure across all environmental pathways [38,39]. Public awareness campaigns about the sources and risks of heavy metal exposure can also significantly reduce individual exposure [40]. Additionally, interventions to reduce chronic stress, such as stress management programs, mental health services, and community support systems, should be integrated into public health strategies. Promoting lifestyle changes that reduce stress, such as regular physical activity, healthy eating, and sufficient sleep, can also help mitigate the impact of chronic stress on lipid profiles [41]. Regular screening for heavy metal exposure and stressors in populations at risk can help identify individuals who might benefit from targeted interventions [42]. Incorporating these measures into routine health check-ups can facilitate the early detection and management of lipid abnormalities.

### 4.1. Limitations

While this study has several strengths, including the use of comprehensive NHANES data and advanced structural equation modeling, there are limitations to consider. The cross-sectional nature of NHANES data limits the ability to infer causality. Longitudinal studies are needed to establish temporal relationships and causal pathways. Future research should focus on longitudinal studies to establish causal relationships between heavy metal exposure, allostatic load, and lipid profiles and explore these factors’ long-term impact on cardiovascular health. Mechanistic studies investigating the biological mechanisms through which heavy metals and chronic stress influence lipid metabolism could provide deeper insights and identify potential therapeutic targets. Finally, another limitation of this study is the lack of access to geographically specific data from NHANES, as this information is restricted and not publicly available. Geographical variation in exposure to environmental contaminants, particularly in economically depressed areas such as Flint, MI, could provide important insights into the relationship between pollutant exposure and cardiovascular risk. Future studies that combine NHANES data with geographically specific datasets may help to uncover the socio-economic and geographical disparities in exposure and related health outcomes. 

### 4.2. Future Work

Although our study focused on the association between heavy metals (lead and cadmium), allostatic load, and lipid dysregulation in relation to cardiovascular disease risk, it is important to acknowledge that other essential elements, such as calcium (Ca^2+^), potassium (K^+^), and magnesium (Mg^2+^), may also play significant roles in cardiovascular health. Future research could explore the interactions between these essential elements and cardiovascular outcomes to provide a more comprehensive understanding of how both toxicants and essential elements contribute to cardiovascular disease risk. Incorporating these elements into future analyses could complement our findings and offer additional insights into potential protective factors against cardiovascular dysregulation.

## 5. Conclusions

This study provides compelling evidence for the significant impact of environmental pollutants and physiological stress on lipid profiles, key markers of cardiovascular health. Utilizing data from NHANES 2017–2018 and advanced structural equation modeling, we demonstrated that elevated levels of lead and cadmium, along with higher AL scores, are associated with unfavorable lipid profiles. Specifically, increased exposure to these heavy metals and higher physiological stress levels were linked to higher LDL and triglyceride levels, as well as lower HDL levels.

Our findings underscore the multifactorial nature of CVD risk, highlighting the critical roles that environmental exposures and chronic physiological stress play in lipid metabolism and cardiovascular health. The observed associations suggest that public health strategies should focus not only on reducing heavy metal exposure but also on mitigating the effects of chronic stress to effectively address CVD risk.

These results pave the way for further research into the mechanisms underlying these associations and the development of targeted interventions. Addressing both environmental and physiological factors in public health policies could lead to more comprehensive and effective strategies for reducing the burden of cardiovascular diseases.

## Figures and Tables

**Table 1:** Mean levels of study variables of interest.

	Mean	SE
**Lead**	1.0684146	0.0335956
**Cadmium**	0.4306898	0.0174229
**AL**	3.5000254	0.0580942
**LDL**	108.6576531	1.4042796
**Triglycerides**	102.3661472	1.9408631
**HDL**	54.3415443	0.7188589
**Age**	44.3281270	0.6236168

**Table 2:** Structural equation model of metals, AL, and LDL cholesterol.

Path	Estimate	Std. Error	z-Value	*p*-Value	Std. Estimate
**Metals = ~z_Lead**	0.531	0.187	2.833	0.005	0.468
**Metals = ~z_Cadmium**	0.391	0.139	2.819	0.005	0.355
**z_LDL~Metals**	0.087	0.036	2.431	0.015	0.087
**z_LDL~z_AL**	0.222	0.019	11.701	0.000	0.220
**z_LDL~z_Age**	0.128	0.023	5.539	0.000	0.104

**Table 3:** Structural equation model of metals, AL, and HDL cholesterol.

Path	Estimate	Std. Error	z-Value	*p*-Value	Std. Estimate
**Metals = ~z_Lead**	0.224	0.234	0.958	0.338	0.198
**Metals = ~z_Cadmium**	0.921	0.958	0.962	0.336	0.836
**z_HDL~Metals**	0.045	0.050	0.893	0.372	0.044
**z_HDL~z_AL**	−0.346	0.019	−18.319	0.000	−0.334
**z_HDL~z_Age**	0.146	0.023	6.310	0.000	0.115

**Table 4:** Structural equation model of metals, AL, and triglycerides.

Path	Estimate	Std. Error	z-Value	*p*-Value	Std. Estimate
**Metals = ~z_Lead**	0.559	0.380	1.472	0.141	0.494
**Metals = ~z_Cadmium**	0.369	0.251	1.469	0.142	0.335
**z_TG~Metals**	0.044	0.035	1.263	0.207	0.044
**z_TG~z_AL**	0.374	0.018	20.595	0.000	0.368
**z_TG~z_Age**	0.115	0.022	5.145	0.000	0.092

## Data Availability

The NHANES dataset is publicly available online, accessible at https://wwwn.cdc.gov/nchs/nhanes/continuousnhanes/overview.aspx?BeginYear=2017 (accessed on 22 December 2023).
